# The effects of purchasing alcohol and marijuana among adolescents at-risk for future substance use

**DOI:** 10.1186/1747-597X-9-38

**Published:** 2014-09-18

**Authors:** Karen Chan Osilla, Eric R Pedersen, Brett A Ewing, Jeremy NV Miles, Rajeev Ramchand, Elizabeth J D’Amico

**Affiliations:** RAND Corporation, 1776 Main Street, P.O. Box 2138, Santa Monica, CA 90407-2138 USA; RAND Corporation, 1200 South Hayes Street, Arlington, VA 22202-5050 USA

**Keywords:** Adolescents, Drug market, Marijuana, Alcohol and drug outcomes, Substance use, Purchasing

## Abstract

**Background:**

Among high-risk youth, those who may be at increased risk for adverse alcohol and other drug (AOD) use outcomes may benefit from targeted prevention efforts; how youth acquire AOD may provide an objective means of identifying youth at elevated risk.

**Methods:**

We assessed how youth acquired alcohol and marijuana (purchasing vs. other means), demographics, AOD behaviors/consequences, and environment among adolescents referred to a diversion program called Teen Court (N = 180) at two time points (prior to the program and 180 days from baseline). Participants were predominantly White and Hispanic/Latino(a).

**Results:**

In cross-sectional analyses among alcohol and marijuana users, purchasing marijuana was associated with more frequent marijuana use and consequences, time spent around teens who use marijuana, higher likelihood of substance use disorders, and lower resistance self-efficacy compared to non-purchasers. Teens who purchased both alcohol and marijuana experienced similar outcomes to those who purchased only marijuana, and also reported more frequent and higher quantity of drinking, greater alcohol-related consequences, time spent around teens who use other drugs, and prescription drug misuse. Longitudinally, purchasing alcohol and marijuana at baseline was associated with more frequent and higher quantity of drinking compared to non-purchasers at follow-up. Marijuana only purchasers had a greater likelihood of substance use disorders at follow-up compared to non-purchasers.

**Conclusions:**

In an era where drinking is commonplace and attitudes towards marijuana use are becoming more tolerant, it is essential to evaluate how accessibility to AOD and subsequent purchasing behaviors affect youth consumption and intervene accordingly to prevent future consequences.

## Background

Adolescence is an important developmental period when many youth may gain access to alcohol and other drugs (AODs). During late middle school and early high school (approximately ages 13 to 16), many youth initiate alcohol and marijuana use [1–3]. By 8^th^ grade 33% of adolescents report having initiated drinking and 16% report marijuana initiation; figures that jump to 54% for alcohol initiation and 34% for marijuana initiation by the 10^th^ grade [4]. Early initiation of substances can lead to a host of current and future substance use problems such as poor school performance, school drop-out and delinquency, health problems, and future risk for substance use disorders [5–9].

Increased access to substances during adolescence may be a contributory factor to initiation. Over the past 35 years, many adolescents report that alcohol and marijuana are easy to obtain. In 2012, 78% of 10^th^ graders and 91% of 12^th^ graders reported easy access to alcohol, and 69% of 10^th^ graders and 82% of 12^th^ graders reported it would be easy to obtain marijuana [4]. Policies that aim to reduce access to alcohol, in particular, by holding merchants liable for selling to underage minors and restricting the hours of sales have been effective in changing merchant practices [10], yet further evidence is needed to demonstrate the public health impact of these efforts on youth purchasing and AOD using behaviors. A multifaceted approach is likely needed to ultimately reduce adolescent AOD use. For example, it may be important to hold commercial vendors more accountable for their actions or establish stricter sanctions when someone who purchases substances legally (e.g., through a medical marijuana card) sells it to someone else. However, it may be equally important to screen and identify youth who purchase AOD and discuss the potential effects of purchasing with them to prevent future consequences. Little research has addressed the purchasing behavior of youth and more research is needed to best inform policies and interventions targeted toward both youth and their suppliers. Thus, we designed the present study to provide a better understanding of youth purchasing behavior of the two most popular drugs among adolescents: alcohol and marijuana.

### Alcohol

Alcohol is accessible to youth from a variety of sources. Alcohol is most often obtained by adolescents from social sources, including peers and family members. Provision by friends over the age of 21 is the biggest contributor to alcohol use among teenagers [11–14], but youth also report provision by parents and guardians [15]. Although the majority (78%) of 15 to 17 year-olds report that they did not pay for the alcohol during their last use [16], 30% report having purchased alcohol from commercial sources [11]. Four out of five teenagers who bought alcohol asked someone else to purchase the alcohol for them, with the remaining 20% purchasing it themselves from a store, restaurant, or bar [16].

Youth who purchase alcohol through commercial sources may experience greater rates of drinking and alcohol-related problems compared to those who obtain alcohol solely through social sources. Studies have found that youth who purchase alcohol drink more frequently, are more likely to report heavy drinking, and are more likely to report drinking and driving, riding in a car with someone who was drinking, and drinking on school property [11, 12, 17]. Though these studies are limited by cross-sectional data, findings suggest that there may be an association between purchasing alcohol and heavier drinking and consequences.

### Marijuana

Similar to alcohol, most adolescents obtain marijuana through social sources like friends, yet studies evaluating the purchasing sources of marijuana among youth are limited. About 57% of youth aged 12 to 17 who use marijuana typically report obtaining the drug for free, though 36% report purchasing marijuana for their most recent use [16]. This pattern may be different for youth who are at-risk (i.e., those adjudicated for AOD-related offenses). For example, in a large study of teenagers involved in the court system, the majority of teens said that they obtained marijuana themselves (58%), as opposed to being given the drug by a peer (18%) or having a friend or relative obtain the drug (7%) [18].

It may be assumed that adolescent purchasers of marijuana are also at increased risk for consequences compared to those who receive the drug from social sources; however, there is little research examining the risks associated with purchasing marijuana among youth. Existing literature focuses on purchases of illicit drugs more broadly, making it difficult to disentangle the characteristics and consequences of teens who only purchase marijuana. Consistent with the characteristics of teens who purchase alcohol, teens who purchase illicit drugs are also more likely to be male, older, and report heavier marijuana use [17]. No research to our knowledge has compared the effects of purchasing and not purchasing marijuana on subsequent consequences. Given elevated rates of marijuana use among adolescents [4], increased access to marijuana for medical use in some states [19], and growing perceptions among youth that marijuana use is beneficial or “normal” [20–22], it is important to understand how purchasing of marijuana during this developmental period may affect subsequent use. Any method of identifying risk and effectively intervening could have high impact in preventing future AOD problems for these youth in adulthood. Furthermore, this research is important as teens that purchase marijuana tend to do so from individuals they just met or from strangers, and youth who purchased marijuana are also more likely to purchase it in a public setting versus in a home [16, 18]. How youth obtain marijuana may increase the probability of being arrested and experiencing other legal consequences [23].

### Purchasers of both alcohol and marijuana

There is currently no research comparing the risks of purchasing both alcohol and marijuana versus purchasing only one substance. More teenagers buy marijuana than alcohol (36% versus 22%) [16] and it is possible that those who purchase marijuana may also be the same youth who are purchasing alcohol. Teens report that marijuana is the easier of the two drugs to buy [24] and when teens use marijuana, they are often also consuming alcohol [25–27]. Adolescents and young adults who use both alcohol and marijuana may be at increased risk for heavier use and consequences than those who use only one substance [28–30]. However, there is a substantial gap in the literature on examining purchasers of *both* alcohol and marijuana.

### The present study

This study focuses on adolescents who reported use of alcohol or marijuana. We compare youth who purchased and did not purchase alcohol and marijuana by examining associations between purchasing and youth demographic characteristics, AOD use behaviors and consequences, and the adolescent’s environment in both cross-sectional and longitudinal analyses. As the few available studies on purchasing behavior have been cross-sectional and limited to school-based samples with descriptive information only, we utilize longitudinal data from an at-risk sample involved in the California Teen Court system to identify the correlates and consequences of purchasing alcohol, marijuana, and purchasing both substances. We hypothesized that youth who purchased alcohol and/or marijuana would report greater AOD use, consequences, and environmental risk compared to youth who did not purchase, and that purchasing both alcohol and marijuana would lead to greater risk than purchasing only one substance. Youth who are involved in the juvenile court system for a first time AOD offense are at-risk for both delinquent behavior (e.g., skipping school; [31]) and heavy substance use [32]. Thus, understanding purchasing behavior among these adolescents and how it relates to subsequent substance use can help inform interventions and policy directed toward identifying youth at risk for more intensive interventions.

## Methods

### Consent

Study protocols were approved by RAND’s Institutional Review Board. Parents were required to provide written consent for their adolescent to participate (if they were under 18) and youth had to provide written assent (if under the age of 18) or consent (if 18).

### Setting

This longitudinal study was part of a larger randomized controlled trial examining the effects of a group-based Motivational Interviewing intervention, *Free Talk*, compared to usual care intervention groups in a Teen Court diversion program [33]. The program is offered to adolescents who commit a first-time AOD offense and are not deemed in need of more serious intervention by the local probation department. Teens complete six group sessions and additional requirements (e.g., community service) in order to have their AOD offense expunged from their probation records.

### Participants

Youth referred to Teen Court between 2009 and 2011 who met inclusion criteria (i.e., first-time AOD offense; 14–18 years old; and English proficient) and did not meet exclusion criteria (i.e., referral to another program; possession of a medical marijuana prescription card; or multiple offenses) were invited to participate. Of those eligible (n = 216), 23 were either not interested or unable to participate and 12 reported no use of alcohol in the past year or drugs in their lifetime. One participant was excluded for missing data, leaving a sample of 180. Only four of the 180 teens dropped out of the Teen Court program, but we were able to obtain follow-up data on three of these teens. Marijuana infractions made up 38% of cases, with the remainder of youth cited for concurrent alcohol and marijuana use infractions (4%) or other drug offenses (2%). More youth (57%) reported marijuana as their drug of choice compared to alcohol (43%). There were no significant demographic (age, gender) or offense differences between refusers/non-participants and participants. Compared to the general population in California, our sample consisted of more male, Hispanic/Latino(a), and white youth [34].

### Procedures

#### Data collection

Youth completed a survey administered by trained staff before they started their Teen Court program and completed another survey approximately 180 days from the time of the baseline interview. Participants were paid $25 for baseline and $45 for the follow-up. Both surveys were completed in a private room at the location where AOD groups were held.

### Measures

#### Demographics

Demographic information included age, gender, and race/ethnicity (Hispanic/Latino(a), White, Other), and offense (alcohol; marijuana; other) information collected from court records.

Mental health symptoms (anxiety, positive affect, depression, and emotional control) were assessed with the five-item Mental Health Inventory (MHI-5) [35]. Participants indicated the frequency to which they experienced five symptoms on a scale from “1 = all the time” to “6 = never”. The two general positive affect items were reversed coded and summed with higher scores indicating better mental health (α = 0.82).

#### Sources of alcohol and marijuana

Using items from the National Survey on Drug Use and Health [16], participants reported at baseline whether or not they had paid for alcohol in the past 12 months. These participants were classified as drinkers who purchased alcohol. Participants who had consumed alcohol but did not purchase it (someone else gave it to them, or they took it from their own or someone else’s home) were classified as drinkers who had not purchased alcohol.

Participants were also asked about the last time they bought marijuana. Those who used marijuana and endorsed ‘I have not bought marijuana in the past 12 months’ were classified as non-purchasers. Marijuana purchasers endorsed ‘I bought it from a friend’, ‘I bought it from a family member’ or ‘I bought it from someone I had just met or didn’t know well’ [16].

### Outcomes

#### Alcohol use and consequences

Past 30 day drinking, including heavy drinking (5+ drinks within a few hours), was assessed using an 8-point scale to indicate the number of days used (1 = ‘0 days’ to 8= ’21-30 days’). Participants were also asked how many drinks on average they consumed on any given occasion in the past 30 days and the maximum number of drinks on any occasion in the past 30 days. Drinks per drinking day was rescaled from an 8-point scale (1 = ‘Never’ to 8 = ‘more than 12’) to a pseudo-continuous variable that ranged from 0 to 12 using the mid-point of any drink range as the new value (e.g., 3–4 drinks was recoded as 3.5 drinks). Maximum number of drinks was coded on a 25 point scale (0 = ‘0 drinks’ to 25 = ‘25 or more drinks’). Six items rated on a 4-point scale (0 = ‘Never’ to 3 = ‘3 or more times’) assessed negative alcohol consequences (α = 0.67) in the past 30 days [36]. Items were summed with a possible total ranging from 0 to 18, with higher scores indicating experiencing more severe consequences.

#### Marijuana use and consequences

Marijuana use was assessed using an 8-point scale to indicate the number of days used (1 = ‘0 days’ to 8=’21-30 days’). Five items assessed marijuana consequences (α = 0.69) in the past 30 days [36]. This scale was rated on a 4-point scale (0=’Never’ to 3=’3 or more times’) and items were summed with a possible total score ranging from 0 to 20, with a higher score indicating more severe consequences.

#### Prescription drug use

Past 30 day prescription drug use was dichotomized due to the highly skewed distribution (0 = no use, 1 = any use).

#### Substance use disorder

To evaluate the severity of substance use, we asked five substance problem scale items from the GAIN short screener and determined threshold of substance use disorder [37]. Questions (e.g., “you spent a lot of time either getting alcohol or marijuana, using alcohol or marijuana, or feeling the effects of alcohol or marijuana”) were asked about the past 30 days, past 2–12 months, or 1 or more years ago. The number of times a participant endorsed that the items occurred in the past year were summed (range 0–5) and then categorized into three diagnostic categories: Low (0 items endorsed; unlikely to need services), Moderate (1–2 items endorsed; a possible substance use disorder and are likely to benefit from a brief intervention) or High (3 or more items endorsed; high probability of a disorder and need for more formal assessment and intervention).

#### Intentions to use and resistance self-efficacy (RSE)

Participants were asked whether they would drink any alcohol or use any marijuana in the next six months. Responses for each item ranged from 1 (definitely use) to 4 (definitely not use). The items were reverse coded so that a higher value indicated greater intentions to use. RSE of alcohol and marijuana were each assessed using 4 items that asked whether the participant would use alcohol or marijuana if their best friend was using, if they were bored at a party, if all their friends at a party were using, or if their boyfriend or girlfriend was using [38, 39]. Responses ranged from 1 (I would definitely drink/use) to 4 (I would definitely not drink/use). The items were summed to create a score where a greater value indicates higher self-efficacy (alcohol α = 0.91; marijuana α = 0.96).

#### Time spent around teens who use

One question, rated on a 4-point scale (1 = ‘Never’ to 4 = ‘Often’) asked about time spent around teens that use alcohol [36]. Two more questions asked about time spent around teens that use marijuana or other drugs (e.g., cocaine, methamphetamine), respectively.

### Statistical analyses

We first examined descriptive statistics for those who purchased alcohol only, those who purchased marijuana only, and those who purchased both alcohol and marijuana. Because of the small percentage of alcohol-only purchasers in our sample (n = 14), we excluded alcohol-only purchasers and only examined statistical differences between non-purchasers and those who purchased marijuana only, and non-purchasers and those who purchased both alcohol and marijuana.

We then divided the sample into those who used marijuana and those who used both marijuana and alcohol and examined the cross-sectional association between purchasing and demographic, AOD- related behaviors and attitudes. We conducted these comparisons using chi-square and t-tests. We also conducted regression analyses to evaluate the longitudinal association between the three types of purchasing behavior at baseline (marijuana only, alcohol and marijuana and non-purchasers) and AOD-related behaviors and attitudes at follow-up. Using non-purchasers as our reference group, we created two dummy coded indicator variables to represent each of the purchaser groups (i.e. marijuana only, marijuana and alcohol). These indicators were included in a model that also controlled for age and group intervention assignment. Mean and modal imputation was used to account for the minimal amount of missing data (<1%) within scales.

## Results

### Descriptive analyses

#### Overall sample

Participant mean age was 16.7 (SD = 1.05). Sixty-seven percent of teens were male, 43.4% identified as Hispanic or Latino(a), 46.1% non-Hispanic white and 10.6% reported another race (e.g., mixed, Pacific Islander).

#### Alcohol-only purchasers

Of the 180 teens in the sample, 159 teens reported any alcohol use at baseline (16 reported alcohol only, 143 reported alcohol and marijuana use). Of these 159 teens, 14 teens reported purchasing alcohol at baseline. All said they gave money to someone else to buy the alcohol. The mean age for the alcohol-only purchasers was 17.2 (SD = 1.2). Seventy-one percent were male, 29% identified as Hispanic or Latino(a), 57% white and 14% reported another race. About 93% of these teens had an alcohol-related offense. Eighty-six percent of the alcohol-only purchasing teens reported alcohol as their most used substance and 21.4% reported often spending time around teens who used alcohol.

#### Marijuana-only purchasers

Of the 180 teens, 164 teens reported marijuana use at baseline (21 reported marijuana only, 143 reported alcohol and marijuana use). Of these 164 teens, 78 (47.6%) reported purchasing marijuana and not alcohol. Most marijuana-only purchasers bought it from a friend (73.1%); the remainder bought it from someone they just met or did not know well.

#### Dual substance purchasers

Of the 143 teens who reported both alcohol and marijuana use at baseline, 37 teens (25.9%) reported purchasing both alcohol and marijuana. Most teens reported giving money to someone to purchase alcohol (86.5%). Five teens (13.5%) said they purchased alcohol for themselves. These dual-purchasers mostly purchased marijuana from a friend (75.7%), followed by someone they just met or didn’t know well (21.6%), and one teen purchased marijuana from a family member/relative.

### Baseline differences

#### Marijuana purchaser characteristics

Among marijuana users, marijuana-only purchasers and non-purchasers were similar in demographics (Table 1). Youth who did not purchase marijuana were more likely to have had an alcohol offense and reported alcohol as their most used substance compared to those who purchased marijuana. Marijuana-only purchasers reported significantly more days of marijuana use in the past 30 days, more marijuana-related consequences, more time spent around teens who use marijuana, and greater intentions to use marijuana compared to non-purchasers. Marijuana-only purchasers were also more likely to be classified by the GAIN as having a high probability of a substance use disorder and needing more formal assessment compared to non-purchasers. Resistance self-efficacy for both alcohol and marijuana use was significantly lower for marijuana-only purchasers compared to non-purchasers.

**Table 1:** **Sample characteristics by status^**

	Marijuana user				Marijuana and alcohol user			
	Purchased MJ only	Non-purchaser	Test statistic**	P- value	Purchased alcohol & MJ	Non-purchaser	Test statistic**	P- value
	n = 78	n = 49			n = 37	n = 27		
	Mean (SD)/%	Mean (SD)/%			Mean (SD)/%	Mean (SD)/%		
Demographics								
Age	16.5 (1.0)	16.7 (1.1)	1.35	0.180	17.0 (1.0)	16.9 (0.8)	0.58	0.566
Male	64.1	69.4	0.38 (1)	0.540	70.3	59.3	0.84 (1)	0.360
Race			0.74 (2)	0.690			2.97 (2)	0.227
Hispanic/Latino(a)	42.3	34.7			54.1	33.3		
White	47.4	53.1			35.1	55.6		
Other	10.3	12.2			10.8	11.1		
Alcohol offense	38.5	65.3*	8.68	0.003	59.5	74.1	1.48 (1)	0.224
MHI-5 (0–100)	72.4 (15.6)	69.9 (20.3)	0.80	0.427	65.6 (20.2)	69.4 (20.1)	0.74	0.461
Alcohol use & consequences								
Alcohol in the past 30 days	2.5 (1.6)	2.2 (1.4)	1.00	0.321	3.5 (1.6)	2.4 (1.1)*	3.15	0.003
Heavy drinking in the past 30 days	2.0 (1.6)	1.6 (1.2)	1.30	0.197	2.5 (1.8)	1.5 (1.1)*	2.61	0.011
Drinks per drinking day	2.5 (3.1)	1.9 (2.4)	1.08	0.283	3.8 (2.9)	2.1 (1.7)*	2.79	0.007
Number of drinks on max occasion	3.4 (4.4)	2.9 (4.1)	0.66	0.510	6.9 (6.2)	2.9 (3.1)*	3.11	0.003
Alcohol most used substance	20.5	60.4*	20.61 (1)	<.001	43.2	70.4*	4.64 (1)	0.031
Alcohol-related consequences	1.3 (0.5)	1.2 (0.3)	1.34	0.184	1.4 (0.4)	1.1 (0.2)*	2.88	0.005
Time spent around teens who drink	2.6 (0.9)	2.6 (0.9)	0.10	0.923	3.1 (0.8)	2.8 (0.7)	1.83	0.072
Marijuana or other drug use & consequences								
Marijuana use in the past 30 days	4.1 (2.3)	2.1 (1.6)*	5.10	<.001	3.8 (2.5)	2.5 (1.9)*	2.35	0.022
Marijuana-related consequences	1.3 (0.5)	1.1 (0.4)*	2.03	0.045	1.3 (0.5)	1.2 (0.5)	0.98	0.329
Time spent around teens who use marijuana	3.1 (0.8)	2.6 (0.9)*	2.45	0.016	3.2 (0.8)	2.9 (0.8)	1.35	0.182
Any Rx drug use past 30 days (%)	6.4	0.0		0.156	21.6	0.0*		0.017
Time spent around teens who use other illegal drugs	1.6 (0.9)	1.5 (0.8)	0.72	0.472	2.1 (1.0)	1.5 (0.8)*	2.46	0.017
GAIN past year Intentions to use	1.0 (0.7)	0.4 (0.6)*	5.08	<.001	1.2 (0.6)	0.4 (0.6)*	5.70	<.001
Intention to use alcohol	2.3 (0.9)	2.0 (0.8)	1.96	0.052	2.6 (1.0)	2.1 (0.6)*	2.44	0.018
Intention to use marijuana	2.2 (1.1)	1.5 (0.8)*	3.96	<.001	2.3 (1.0)	1.6 (0.7)*	2.87	0.006
Resistance self-efficacy								
Resistance self-efficacy - alcohol	10.7 (3.5)	12.8 (3.2)*	-3.37	0.001	10.8 (3.4)	12.0 (3.1)	1.39	0.169
Resistance self-efficacy - marijuana	10.9 (4.2)	14.3 (2.4)*	-5.27	<.001	11.4 (4.1)	13.8 (2.8)*	2.57	0.013

*p<0.05; ^27 respondents were classified as both a marijuana user, non-purchaser, and a marijuana and alcohol user, non-purchaser. **Reported test statistics are from t-tests for continuous variables and chi-squared tests for dichotomous and categorical variables. Only for prescription drug use did we use a Fisher’s exact test due to small cell counts. For this test only the p-value is reported. Degrees of freedom for marijuana user t-tests are 125 and the degrees of freedom for the dual purchaser t-tests are 62. Degrees of freedom for chi-squared tests are indicated in parentheses after the test statistic.

#### Marijuana and alcohol purchaser characteristics

Among alcohol and marijuana users, dual-purchasers and non-purchasers were similar in demographics (Table 1). Dual-purchasers were significantly less likely to report alcohol as their most used substance compared to non-purchasers, although dual-purchasers reported significantly more days of alcohol use, more heavy drinking, more drinks per drinking day, more drinks on the occasion when they drank the most in the past 30 days, and more alcohol related consequences compared to non-purchasers. Compared to non-purchasers, dual-purchasers reported more days of marijuana use, more time spent around teens who used other illegal drugs than non-purchasers, greater intentions to use alcohol and marijuana, lower resistance self-efficacy for marijuana, and were also more likely to report prescription drug misuse. Furthermore, dual-purchasers were more likely to be classified by the GAIN as having a high probability of a substance use disorder and needing formal assessment.

### Regression analyses

#### Marijuana-only, dual-purchaser, and Non-purchaser outcomes

Table 2 shows that there were significant differences at the follow up in past month alcohol use between teens who purchased both alcohol and marijuana, teens who purchased marijuana only, and non-purchasers. Alcohol use differed significantly between the three groups, with dual-purchasers increasing the most on the frequency and quantity of typical and heavy drinking occasions compared to the other two groups.

**Table 2:** **Effects of purchasing marijuana, both alcohol and marijuana, or neither at baseline on follow-up outcomes†**

	Purchased only marijuana	Purchased alcohol & marijuana	Non-purchaser	F (df = 2, 153)	P
	(n = 76)	(n = 33)	(n = 49)		
	Mean/SD (%)	Mean/SD (%)	Mean/SD (%)		
Alcohol use & consequences					
Any alcohol in the past 30 days^	2.36 (1.44)	3.61 (1.58)	2.63 (1.52)	5.98	0.003
Heavy drinking in the past 30 days	1.71 (1.29)	2.58 (1.73)	1.65 (1.28)	4.22	0.017
Drinks per drinking day	2.24 (2.75)	3.98 (2.88)	2.07 (2.06)	5.73	0.004
Number of drinks on max occasion	3.55 (4.84)	7.09 (6.04)	3.63 (4.62)	5.07	0.007
Alcohol-related consequences^^	1.08 (1.83)	1.12 (1.82)	0.78 (1.43)	0.75	0.472
Time spent around teens who drink	2.75 (0.88)	3.24 (0.87)	2.94 (0.85)	2.51	0.085
Marijuana or other drug use & consequences					
Any marijuana use in the past 30 days	3.04 (2.35)	3.09 (2.38)	2.27 (1.78)	2.43	0.091
Marijuana-related consequences	0.72 (1.65)	1.15 (1.72)	0.41 (1.21)	2.21	0.113
Time spent around teens who use marijuana	3.03 (0.86)	3.15 (0.94)	3.10 (0.96)	0.01	0.995
Prescription drug misuse	7.89	12.12	6.12	1.51	0.541
Time spent around teens who use other illegal drugs	1.79 (0.96)	2.00 (1.09)	1.86 (0.94)	0.42	0.658
GAIN past year	1.07 (0.70)	1.00 (0.79)	0.78 (0.62)	3.14	0.046
Intentions to use					
Intention to use alcohol	2.68 (1.02)	2.91 (0.98)	2.55 (0.89)	1.38	0.255
Intention to use marijuana	2.57 (1.02)	2.45 (1.12)	2.04 (1.06)	4.35	0.015
Resistance self-efficacy					
Resistance self-efficacy - alcohol	11.40 (3.61)	11.36 (3.63)	12.16 (3.78)	0.91	0.404
Resistance self-efficacy - marijuana	11.79 (4.05)	11.76 (3.99)	13.29 (3.88)	2.38	0.096

†Models control for age and intervention; ^A mean of 2.36 for any alcohol use in the past 30 days can be interpreted as approximately 1.5 days of use. Similar interpretations can be extended to heavy drinking; ^^A mean consequence score of 1.08 can be interpreted as experiencing approximately one consequence.

Note: The table presents raw means and standard deviations (SD). The reported p-values are the overall p-value for the main effect of purchasing status from the multivariate regression models.

For marijuana use, intentions to use marijuana at follow-up differed significantly between the groups with marijuana only purchasers reporting higher intentions to use marijuana than the other two groups. Substance use severity, as measured by the GAIN score, was also significantly different between the three groups, with non-purchasers having considerably lower scores than the two purchasing groups.

## Discussion

This study examined how alcohol and marijuana purchasing behavior during adolescence affects AOD-related behaviors and attitudes both cross-sectionally and longitudinally. We assessed a diverse sample of at-risk teenagers aged 14–18 who had a first-time AOD offense. About a third of teens reported purchasing alcohol and about 70% reported purchasing marijuana. These numbers are higher than national statistics of teens of similar ages [16] given that we recruited youth from the California Teen Court system with a first-time AOD offense.

The higher proportion of marijuana purchasers compared to alcohol purchasers is noteworthy and may reflect more lenient attitudes around marijuana use. In previous focus group work with at-risk youth, 90% of youth reported positive views about marijuana such as it being less risky and more acceptable compared to other drugs [22]. In the current study, youth often expressed during intervention sessions how they felt marijuana was “natural” and beneficial to the body, and less dangerous than alcohol [21]. These more tolerant attitudes towards marijuana may reflect current trends to legalize marijuana [40], which at the time of this paper was legal in Washington and Colorado. Although it is too early to fully understand the association between legalization, attitudes, and purchasing behavior, this represents a promising area for future research.

There is debate about whether medical marijuana laws increase youth marijuana use [19, 41–44], and the laws in California (where the data were collected) allow use of medical marijuana for adults and adolescents alike. Some research does suggest that areas with more tolerant attitudes about marijuana use (e.g., higher percentage of voters in favor of marijuana legalization) are positively associated with more youth who use marijuana [40]. Additional research suggests that in states that allow medical marijuana, about half of youth in substance use treatment facilities report obtaining marijuana from someone else who uses medical marijuana [45]. As marijuana continues to become more accessible and attitudes about marijuana use become more tolerant, it will be important to regulate and monitor how youth access marijuana and whether their use and subsequent problems escalate. This study excluded youth who possessed a medical marijuana card. Thus, our sample comprised youth who illegally purchased marijuana mostly from friends. Future research is needed to explore where these friends obtained marijuana and whether medical marijuana laws are inadvertently increasing access to marijuana among youth.

There were no demographic differences among youth who purchased marijuana compared to youth who did not purchase marijuana. However, in our cross-sectional analyses, teens who purchased marijuana had greater intentions to use, more recent marijuana use and consequences in the past 30 days, a higher likelihood of a substance use disorder, and lower rates of resistance self-efficacy. Findings highlight that teens who purchase marijuana are at greater risk for future problems. Clinical programs could begin to obtain information about purchasing histories from youth during detailed intake assessments to learn about their potential for risk (e.g., arrests during purchasing) and provide more tailored and intensive intervention for these youth. For example, if marijuana purchasers are more at-risk for subsequent consequences, there may need to be a stronger focus on increasing self-efficacy [46], motivation to change risky behaviors, and building refusal and coping skills to help reduce the effects of substance-related consequences [47]. Interventions could also contain personalized feedback about the amount spent on obtaining substances, provide skills for managing money, and refocus goals of using marijuana to goals associated with saving money to reach a monetary goal (e.g., purchase a new television with the money saved from not buying marijuana). These interventions could build youth’s financial capability to make wise financial decisions, save their money, and plan for their future [48].

Teens who purchased both alcohol and marijuana experienced more severe outcomes compared to teens who purchased marijuana only and non-purchasers. Dual-purchasers appear to be a particularly risky group of teens in terms of future alcohol use. It is likely that a multi-faceted approach is needed to explore the risks associated with purchasing and using both substances, and to curb purchasing of substances by teens. For example, teens might benefit from discussions regarding what might happen if they purchase these substances (e.g., arrests and further legal consequences) [38, 49]. At the policy level, legislation could screen at-risk youth for purchasing and provide more intensive intervention of at-risk youth who report purchasing both alcohol and marijuana. It is also important to work with providers so that they understand the risks associated with purchasing and provide training for providers on how to assess and subsequently intervene. Professionals such as teachers and clinicians can also educate youth on media messages youth are exposed to (including legal medical use and illegal marijuana use) so they have an accurate understanding of the effects of alcohol and marijuana use.

Examining the effects of purchasing on an at-risk sample is critical because these youth are no longer experimenting with use and are already beginning to experience some consequences related to their AOD use. We find in this paper that purchasing is a good predictor of future intentions to use and subsequent substance use disorders. Thus, purchasing behavior may be a possible avenue for identifying teens who are already using AOD and may be at even greater risk for future AOD use problems because of how they acquire these substances. It is important to have screening measures that are easy to administer, do not require extensive time or training, can be incorporated into existing appointments, and that can simply and quickly determine an adolescent’s risk level so that referral and/or treatment services can be provided. However, it is unknown whether purchasing has more predictive power when compared to other severity of use indices such as frequency or quantity and this should be the subject of future research. Overall, results suggest that screening youth for purchasing could be a simple way to assess who may be at greater risk for poorer outcomes, and this could help tailor and potentially increase the efficacy of existing programs for at-risk youth.

All youth in our sample were diverted from treatment and were assigned to receive either six weekly sessions of group-based Motivational Interviewing or usual care Teen Court groups. Our findings suggest that additional treatment may be warranted for youth who purchased alcohol and marijuana as they showed greater use and a higher likelihood of having a substance use disorder compared to non-purchasers, even after receiving an intervention. Thus, multi-faceted interventions for youth with purchasing histories are needed in the juvenile justice system to help reduce future risk by providing more skills to reduce the intentions to purchase, frequency of heavy alcohol use (e.g., harm reduction; [50]), drug use (including prescription drug misuse), and related consequences.

This study addresses an important policy question: what *additional risk* does purchasing alcohol and marijuana have on adolescents who are already using substances and are in the judicial system? Given that purchasing teens reported recent heavier use and consequences and a higher likelihood of a substance use disorder, research is need to determine where teens obtain their alcohol and marijuana, how often these transactions are made, and how these acquisition patterns affect AOD use to fully understand the effects of purchasing behavior [51] and whether interventions can address this behavior.

### Study limitations

Our sample was recruited from one California Teen Court program and may not be representative of at-risk youth nationally. Second, we did not assess purchasing at follow-up so we cannot examine if any changes in purchasing over time were associated with long-term risk. Analyses may also be limited by our small sample size as we were not able to examine the effects of purchasing only alcohol. This limits our ability to determine whether the significant findings found from both purchasing alcohol and marijuana are due to purchasing alcohol or to both substances. All outcomes were collected by self-report. Participants may have felt reluctant to report their use honestly; however, given that youth self-reported substance use on the survey and a large number reported purchasing these substances, we feel confident that the data are accurate.

## Conclusions

For at-risk youth, preventing future substance misuse and criminal justice involvement is essential for changing their life trajectories. Purchasing may be an important and easy to measure behavior that is correlated with future risk, and future research is needed to compare this construct to other validated screening measures. Because youth who engage in delinquent behavior during adolescence also report heavy AOD use and experience consequences from using [32, 52, 53], identifying those who purchase may help identify youth in need of more intensive intervention. If at-risk youth in a Teen Court setting continue to purchase alcohol and marijuana, they may place themselves at greater risk for future incarceration and substance dependence in young adulthood and adulthood. Thus, interventions aimed at reducing purchasing behavior for at-risk youth appear warranted. Interventions established within the Teen Court system may benefit from including discussions of the risks associated with purchasing substances, as well as reinforcing personal values and goals (e.g., not wanting to get in trouble again) to prevent continued substance use. This study is an important first step because laws and beliefs about marijuana are changing. Thus, understanding how youth are accessing AOD and how that affects their future risk is important.
